# Pixantrone demonstrates significant in vitro activity against multiple myeloma and plasma cell leukemia

**DOI:** 10.1007/s00277-019-03797-6

**Published:** 2019-10-18

**Authors:** Ella Willenbacher, Karin Jöhrer, Wolfgang Willenbacher, Brigitte Flögel, Richard Greil, Brigitte Kircher

**Affiliations:** 1 Internal Medicine V (Hematology & Oncology), Medical University of Innsbruck, Anichstr. 35, 6020 Innsbruck, Austria; 2 Tyrolean Cancer Research Institute, Innsbruck, Austria; 3 Oncotyrol, Center for Personalized Cancer Medicine, Innsbruck, Austria; 4 Department of Internal Medicine III, Laboratory for Immunological and Molecular Cancer Research, Paracelsus Medical University Salzburg, Salzburg, Austria

**Keywords:** Multiple myeloma, Plasma cell leukemia, Pixantrone, Anti-myeloma activity, Drug screening

## Abstract

Treatment results for multiple myeloma and plasma cell leukemia have considerably improved, but cure remains elusive and establishing new therapeutic approaches constitutes a major unmet clinical need. We analyzed the anti-myeloma properties of the aza-anthracenedione pixantrone which has been successfully used in a phase III study for the treatment of patients with aggressive non-Hodgkin’s lymphoma as monotherapy as well as in combination regimes in vitro and in an adapted in vivo model (ex ovo chicken chorioallantoic membrane (CAM) assay). Pixantrone significantly inhibited proliferation and metabolic activity of all investigated myeloma cell lines. Importantly, anti-myeloma effects were more pronounced in tumor cell lines than in stromal cells, mesenchymal stem cells, and peripheral blood mononuclear cells of healthy controls. Apoptosis of myeloma cell lines was observed only after a 7-day incubation period, indicating a fast cytostatic and a slower cytotoxic effect of this drug. Pixantrone reduced the viability of primary plasma cells of patients and induced downregulation of myeloma-cell growth in the CAM assay. Additionally, we demonstrate in vitro synergism between pixantrone and the histone deacetylase inhibitor panobinostat with respect to its anti-proliferative features. From these data, we conclude that systematic investigations of the clinical usefulness of pixantrone in the framework of controlled clinical trials are clearly indicated (e.g., in penta-refractory patients).

**Electronic supplementary material:**

The online version of this article (10.1007/s00277-019-03797-6) contains supplementary material, which is available to authorized users.

## Introduction

Multiple myeloma (MM) is an incurable malignant disease of immunoglobulin and/or free light chains producing monoclonal plasma cells, clinically characterized by hypercalcemia, renal failure, anemia, and osteolytic bone disease (so-called CRAB criteria). Considerable clinical progress has been made with respect to higher response rates, better remission-induction efficiency and depth, as well as the improvement of both progression-free and overall survival. However, cure remains an elusive therapeutic goal. Nearly all patients will sooner or later relapse and develop treatment resistance to all available drugs and treatment modalities [1]. Median survival has improved to 7.3 years in cytogenetically defined standard risk patients [2, 3], but can be estimated at less than 2 years in patients harboring combined clinical and genetic risk factors [4, 5]. So-called penta-refractory MM (refractory to bortezomib, carfilzomib, lenalidomide, pomalidomide, and anti-CD38 antibodies) harbors a dismal prognosis with a median survival below 6 months [6].

Plasma cell leukemia (PCL) is a leukemic variant of myeloma arising either de novo or from clinically pre-existent MM in mostly heavily pretreated patients and carries an even worse prognosis than does relapsed/refractory MM. Treatment options are limited, and no drug has ever been explicitly registered for this indication. Furthermore, there is no generally accepted standard of care, although most hematologists use—depending on previous treatments—combinations of MM drugs, steroids, and chemotherapy [7]. Anthracyclines such as doxorubicin (adriamycin) are among the most active classical drugs in lymphoma and myeloma therapy, but their use is limited by cumulative and irreversible cardiotoxicity. With respect to MM therapy, anthracyclines are typically used in combination regimes like bortezomib/adriamycin/dexamethasone (PAD) [8] or bortezomib and pegylated liposomal doxorubicin (B-lipA) [9] and lenalidomide, adriamycin, and dexamethasone (RAD) [10] that combine classical chemotherapy with “novel agents” such as proteasome inhibitors and immune-modulatory drugs (IMiDs).

Pixantrone (PIX) is structurally similar to the anthracycline doxorubicin (Dox), but with an improved toxicity profile, especially with regard to cardiotoxicity [11, 12], while maintaining anti-tumor activity [13, 14]. A phase III clinical trial in massively pretreated aggressive non-Hodgkin’s lymphoma patients led to the drug’s conditional approval in the EU [15]. The classical mode of action of PIX has long been considered to be DNA binding and inhibition of topoisomerase II, an enzyme that is involved in DNA replication [16]. In contrast to classic anthracyclines, PIX has been shown to act selectively on the topoisomerase isoform Iiα [11]. Recently, an additional—quite unique—mechanism of action for inducing cell death, nicknamed induction of “mitotic catastrophe” by mitotic perturbations and subsequent aberrant cell divisions, was described for PIX [17]. Furthermore, a synergistic interaction of proteasome and topoisomerase II inhibition on MM cell lines has been published [18]. These features render the drug a promising candidate, not only for clinical development in aggressive non-Hodgkin’s lymphomas, but also in the still not investigated field of MM.

Here, we investigated the anti-myeloma properties of PIX by (1) analyzing its effects on myeloma cell lines, (2) screening in vitro for anti-myeloma synergisms with putative combination partners, (3) confirming its anti-myeloma effect in a chorioallantoic membrane assay (CAM), and finally (4) generating confirmatory evidence with primary patient material.

## Patients

All patients gave written informed consent to the use of their biologic materials for research (EU FP7 consortium OPTATIO) and documentation of their clinical data via the Austrian Myeloma Registry (AMR) after extensive discussion of the respective procedures.

Patient disposition is outlined in Table 1.

**Table 1:** Patient disposition

Patient	Age (a)	Sex	Disease status	Isotype
1	61	F	Secondary PCL	FLC kappa
2	64	F	Secondary PCL	IgM kappa
3	63	F	Secondary PCL	IgG lambda
4	70	M	PCL	FLC kappa
5	64	M	PCL	IgG kappa
6	49	M	RRMM	IgG lambda
7	70	M	RRMM	IgA kappa

*a*, years; FLC, free light chain; *Ig*, immunoglobulin; *PCL*, plasma cell leukemia; *RRMM*, relapsed/refractory multiple myeloma

## Methods

### Cell culture

Myeloma cell lines (KMS-12-BM, KMS-12-PE, LP-1, RPMI-8226, AMO-1, OPM-2, U-266; purchased from German Collection of Microorganisms and Cell Cultures (DSMZ, Braunschweig, Germany)) and the stromal cell line HS-5 (from ATCC, Manassas, USA) were cultured in RPMI 1640 without phenol red, supplemented with L-glutamine (2 mM), penicillin (100 U/mL), streptomycin (100 μg/mL), and fetal calf serum (FCS, 10% or 20%; all from PAA Laboratories, Pasching, Austria). Cells were cultured at 37 °C in a humidified atmosphere containing 5% CO_2_. The cells were serially passaged twice a week. All cell lines were authenticated by typing short tandem repeats. Mycoplasma contamination was routinely monitored and only mycoplasma-free cultures were used. Mesenchymal stem cells were purchased from PromoCell (Heidelberg, Germany) and cultured in Mesenchymal Stem Cell Growth Medium, according to the manufacturer’s instructions.

### Preparation of peripheral blood mononuclear cells (PBMC) and primary patient material

PBMC from two patients with de-novo PCL, three patients with secondary PCL, and two patients with relapsed/refractory MM as well as four healthy volunteers were isolated by Ficoll density gradient centrifugation. To generate activated PBMC cells (5 × 10^4^) were incubated with 1 μg/mL phytohemagglutinin (PHA; Sigma-Aldrich, Vienna, Austria) and appropriate concentrations of PIX for 72 h.

### Analysis of proliferation

The rate of cell proliferation was measured by labeling with [^3^H]-thymidine as previously described [19]. Proliferation in the absence of the compounds was set at 100%, and the drug’s activity was calculated as percentage of the control (without compound).

### Measurement of metabolic activity

Metabolic activity was determined by measuring the reduction of tetrazolium salts to formazan derivatives using a modified 3-(4,5-di-methyl-thiazol-2-yl)-2,5-diphenyltetrazolium bromide (MTT) assay (EZ4U kit; Biomedica, Vienna, Austria) according to the manufacturer’s instructions. To exclude unspecific staining by FCS-containing medium, the optical density (OD) of the respective medium was subtracted. Metabolic activity in the absence of the compounds was set at 100%.

### Apoptosis assays

Induction of programmed cell death for cell lines and primary cells after different time periods was determined by staining with Annexin V (Ann V)-FITC-labeled antibody and propidium iodide (PI). Samples were further analyzed by flow cytometry (FACS Canto, BD Bioscience, San José, CA, USA) and data were evaluated utilizing FlowJo software (LLC, Ashland, OR, USA).

### Ex ovo chicken chorioallantoic membrane assay (CAM assay)

The CAM assay used in this study was essentially performed as described previously [20]. In detail, collagen onplants containing green fluorescent protein (GFP)-transfected myeloma cells were transferred to opened, fertilized, white leghorn chicken eggs and cultured on their chorio-allantoic membrane with/without the addition of PIX (1 μM). Xenotransplants were microscopically analyzed (Olympus SZX10, Olympus, Vienna, Austria) on day 5. Thereafter, onplants were excised, protein was prepared, and tumor load was determined using anti-GFP ELISA (eBioscience, Thermo Fisher Scientific, Vienna, Austria).

### Statistical analysis

The Wilcoxon rank-sum test was used to analyze the differences between proliferation, metabolic activity, and apoptosis induction in the absence and the presence of the respective concentration of the compounds (NCSS software, Kaysville, UT, USA). A *p* value < 0.05 was considered statistically significant. Additive and synergistic effects of drugs were defined according to the following formula: additive mode of action: surviving fraction (SF) (compound A + compound B) = SF (A) × SF (B); synergistic mode of action: SF (A + B) < SF (A) × SF (B) [21].

## Results

### Effect of PIX on myeloma cell line proliferation

**Fig. 1:**
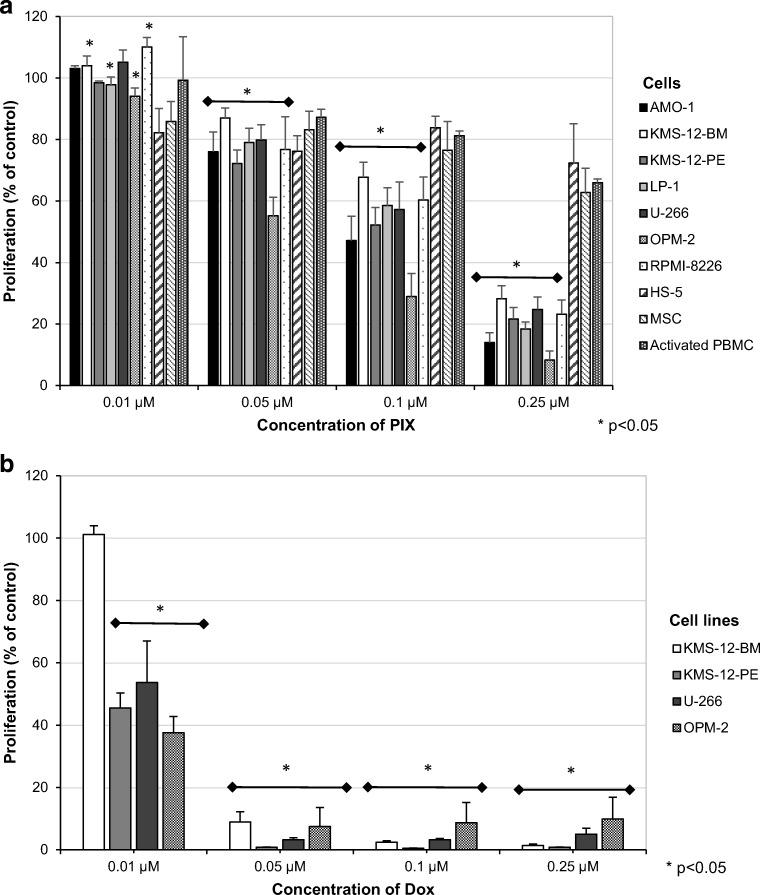
The effect of PIX (**a**) and Dox (**b**) on the proliferation of myeloma cell lines (AMO-1, KMS-12-BM, KMS-12-PE, LP-1, U-266, OPM-2, RPMI-8226), the stromal cell line HS-5, mesenchymal stem cells (MSC) and activated PBMC from four healthy controls determined by [^3^H]-thymidine uptake assay after a 72-h incubation period is shown. Mean proliferation + standard error of at least four experiments is depicted. Proliferation in the absence of PIX and Dox was set at 100%. Statistical significance was determined using the Wilcoxon test (*p < 0.05 against the untreated control)

Proliferation of myeloma cell lines was determined after 72 h of incubation with various concentrations of PIX. All cell lines tested showed reduced proliferation, and the extent was dose-dependent, starting at 0.05 μM PIX (Fig. 1a). At a PIX concentration of 0.25 μM, proliferation was inhibited to 14.0 ± 3.2% (AMO-1, highest responding cell line) and 28.2 ± 4.2% (KMS-12-BM, lowest response). From these data, we assume the IC50 for proliferation inhibition between 0.1 and 0.25 μM. Strikingly, non-MM cells such as the stroma cell line HS-5, mesenchymal stem cells, and PBMC from healthy donors activated with phytohemagglutinin were significantly less affected (average proliferation 67.0 ± 7.3%).

As PIX structurally resembles Dox, a drug still widely used for MM treatment, the anti-proliferative capacity of Dox was analyzed in parallel in selected myeloma cell lines after a 72-h incubation. As shown in Fig. 1b, Dox displayed stronger activity on the myeloma cell lines than PIX. The IC50 for proliferation inhibition was, except for KMS-12-BM, approximately 0.01 μM. After incubation with a concentration of 0.05 μM Dox, almost no proliferation was detected.

Similar anti-proliferative effects of PIX were observed in a co-culture system with the stromal cell line HS-5 (data shown in Supplemental File).

### Effect of PIX on MM cell metabolic activity

**Fig. 2:**
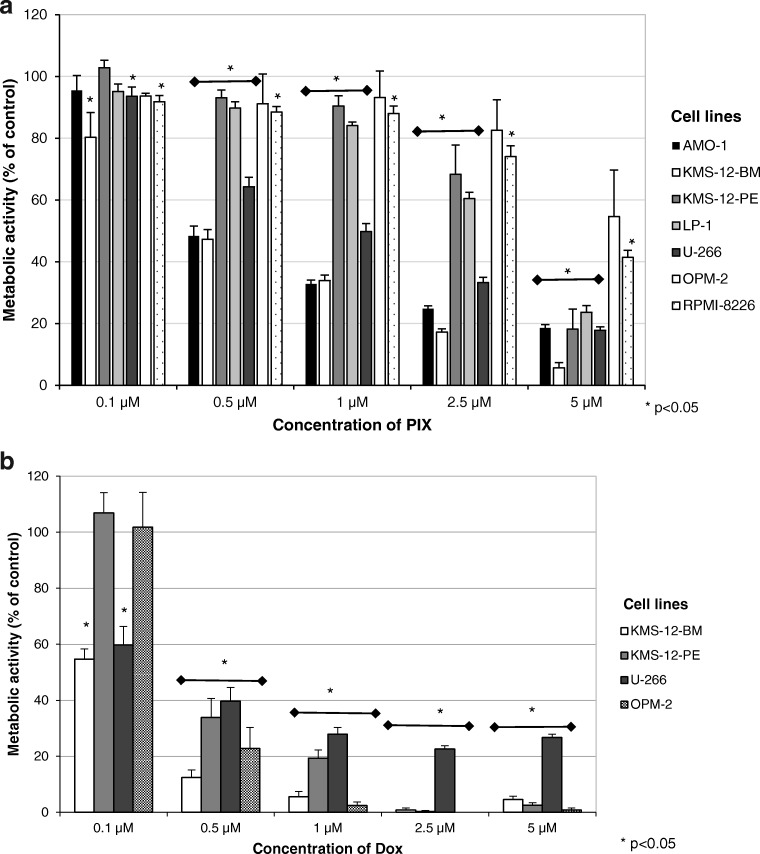
The effect of PIX (**a**) and Dox (**b**) on metabolic activity of MM cell lines after a 72-h incubation period is shown. Mean metabolic activity + standard error of four experiments is depicted. Proliferation in the absence of PIX and Dox was set at 100%. Statistical significance was determined with the Wilcoxon test (*p < 0.05 against the untreated control)

To test whether the strong anti-proliferative activity of PIX resulted also in cytotoxicity, the metabolic activity of mitochondria of the myeloma cell lines was measured after 72 h of incubation with PIX in comparison to Dox. PIX dose-dependently inhibited the metabolic activity of myeloma cell lines (Fig. 2a). The IC50 for the inhibition was cell line-dependent and in the range of 0.5–5 μM. The cell lines AMO-1 and KMS-12-BM (IC50 at 0.5 μM) were more sensitive to PIX treatment than the other cell lines.

Interestingly, also Dox induced cell-line dependent effects in the myeloma cell lines with KMS-12-BM again as the most sensitive cell line and U-266 with the lowest response to Dox treatment (Fig. 2b).

### Effect of PIX on MM cell death

**Fig. 3:**
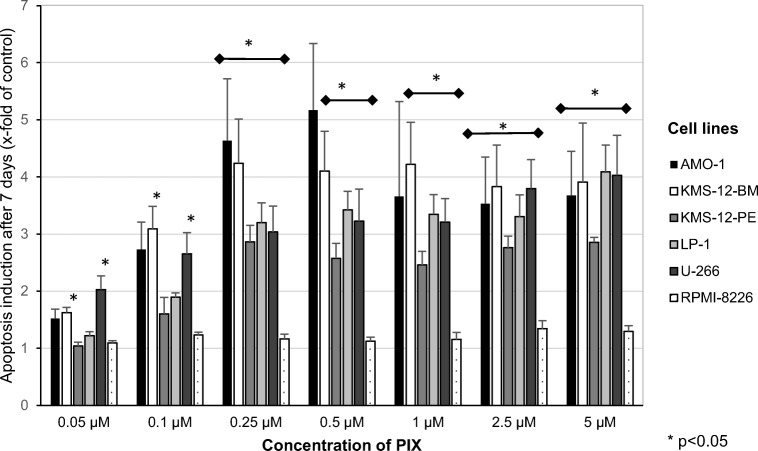
Mean apoptosis induction + standard error of four experiments after an incubation period of 7 days is shown. Apoptosis in the absence of PIX was set at 1. Statistical significance was determined with the Wilcoxon test (*p < 0.05 against the untreated control)

To further evaluate the cytotoxic activity, flow cytometry analyses were performed 48 h and 7 days after PIX treatment. After 48 h, a concentration of 0.25 μM PIX reduced the viability of the cell line KMS-12-BM to 75.3 ± 5.4%, whereas 5 μM decreased it to 45.4 ± 6.7% (data not shown). Apoptosis induction, however, was observed only after a 7-day incubation (Fig. 3).

### Effect of PIX on myeloma cell growth in a CAM assay

**Fig. 4:**
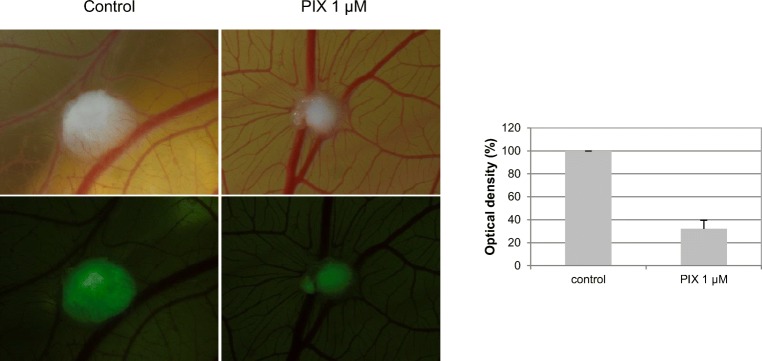
The effect of PIX in an ex ovo chicken chorioallantoic membrane assay is shown. A representative example of the cell growth of the GFP-labeled myeloma cell line OPM-2 after an incubation period of 5 days with and without 1 μM PIX is depicted. Upper view: Transmitted light microscopy, Lower view: GFP staining. Cell growth was determined by GFP ELISA. Mean optical density in the absence of PIX was set at 100%. Mean optical density + standard error of three experiments is depicted

The strong anti-proliferative activity of PIX combined with its cytotoxic behavior led us to further investigate the action of PIX in an adapted in vivo model, the so-called CAM assay, using GFP-labeled OPM-2 cells and a 5-day incubation period. As shown in a representative example in Fig. 4, PIX (1 μM) strongly reduced the growth of the myeloma cells. The tumor load—measured by GFP ELISA—was markedly reduced (35.0 ± 7.5% of control).

### Effect of PIX on MM and PCL cells

**Fig. 5:**
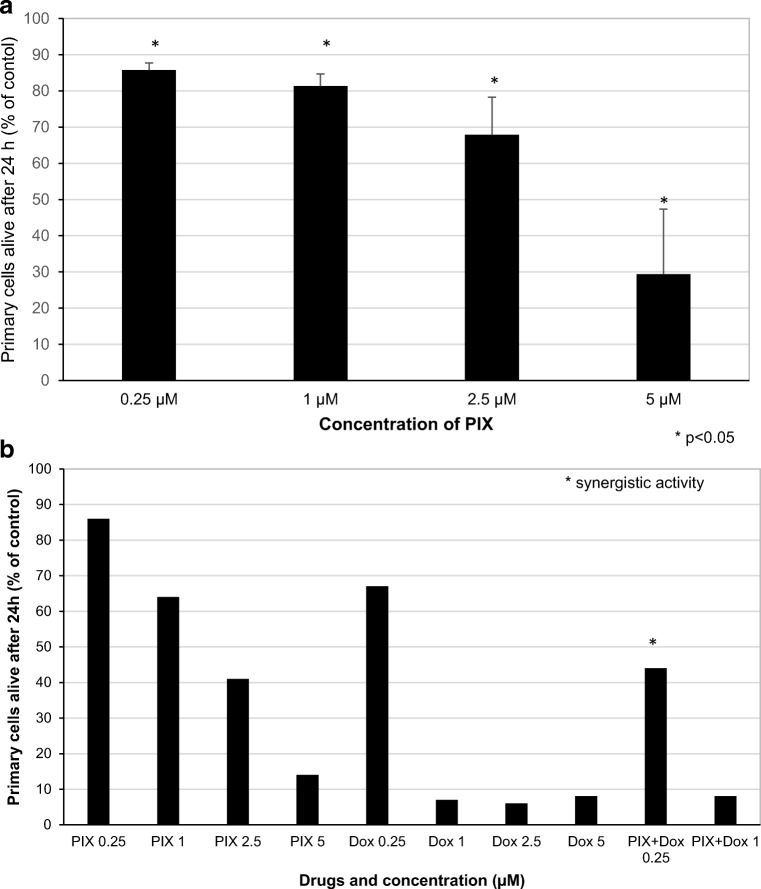
Mean apoptosis induction, as determined by the decline of the percentage of AnnV/PI negative cells by flow cytometry, + standard error of two patients with de-novo PCL, three patients with secondary PCL and two patients with relapsed refractory MM (**a**) after an incubation period of 24 h with PIX is shown. **b** Depicts the percentage of primary cells alive of one patient with relapsed refractory MM incubated for 24 h with PIX and Dox, respectively. Viability of untreated cells was set at 100%. Statistical significance was determined with the Wilcoxon test (*p < 0.05 against the untreated control)

The ability of PIX to induce cell death of primary plasma cells from patients was assessed after a 24-h incubation period in vitro. PIX dose-dependently diminished the extent of the cells alive of two patients with refractory relapsed MM and five patients with de-*novo* or secondary PCL. At 2.5 μM, the proportion of plasma cells alive was reduced to 67.9 ± 10.4% of control without PIX (Fig. 5a).

FACS analysis was further performed with plasma cells of a patient with refractory relapsed MM analyzing the effect of PIX and Dox at various concentrations and in combination (Fig. 5b). PIX dose-dependently reduced the percentage of cells alive, whereas with 1 μM Dox, almost no cells alive were detected any more. At a concentration of 0.25 μM, PIX and Dox displayed synergistic activity.

### Combinatorial activity of PIX

**Fig. 6:**
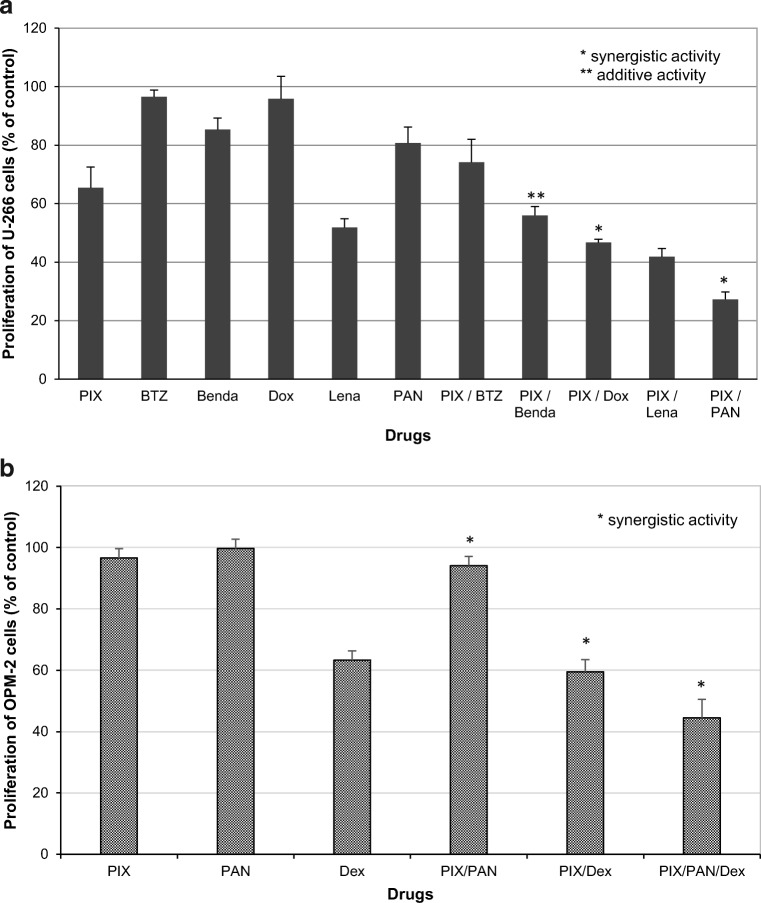
The combinatorial activity of PIX with various anti-MM drugs is depicted. **a** The following compound concentrations were shown: 50 nM for PIX, 5 nM for bortezomib (BTZ), 3 nM for panobinostat (PAN), 10 μM for bendamustine (Benda), 50 nM for doxorubicin (Dox), 10 μM for lenalidomide (Lena). **b** Depicts the following concentrations: 10 nM for PIX, 1.5 nM for PAN, 100 μM for dexamethasone (Dex)

In most cases, MM patients are treated with a combination triple treatment regimen, whereby a combination of even four drugs is also common. We therefore analyzed proliferation of myeloma cells in response to various sublethal concentrations of PIX (10 nM, 50 nM, 100 nM) in combination with various “new” anti-myeloma compounds such as bortezomib (5 and 10 nM), panobinostat (3 nM), and lenalidomide (10 and 25 μM) and “classical” MM drugs such as Dox (10 and 50 nM), bendamustine (10 and 50 μM), and dexamethasone (Dex, 10 and 100 μM). The most effective combination was PIX (50 nM) and panobinostat (PAN; 3 nM) (Fig. 6a). This interesting effect was analyzed in more detail by incubating the cell line OPM-2 with 10 nM PIX, 1.5 nM PAN, and 100 μM Dex (Fig. 6b). Also at lower concentrations, a synergistic effect between PIX and PAN in inhibiting the proliferation of this myeloma cell line was detected. PIX and Dex displayed synergistic activity, and the three drug combination with PAN also induced a proliferation inhibition of OPM-2 cells.

As already shown for primary cells, PIX (50 nM) and Dox (50 nM) revealed also synergistic effects in the myeloma cell lines suggesting additional mechanisms of these drugs. Similar strong anti-proliferative activity was observed with PIX (50 nM) and bendamustine (10 μM).

## Discussion

PIX is approved in the EU for the treatment of aggressive non-Hodgkin’s lymphoma patients. We therefore investigated if PIX might also be effective in MM and PCL.

In all cell lines tested, relatively low PIX concentrations (between 0.1 and 0.25 μM) already significantly inhibited cell proliferation. Importantly, a concentration of 0.25 μM corresponds to plasma levels achievable with standard-dose PIX in patients [22]. This low dosage needed to induce a cytostatic effect suggests that the use of PIX may be feasible as part of combination therapies in MM and PCL patients.

Importantly, anti-myeloma effects were more pronounced in myeloma cell lines than in the stromal cell line HS-5, MSC, and activated PBMC of healthy controls. The results of the CAM assay further support the putative anti-tumor specificity of PIX while inducing no toxic effect on the embryo.

Inhibition of metabolic activity showed a more variant pattern and may reflect the clinical and biological heterogeneity of this disease. The inhibition of metabolic activity after a 3-day culture period was accompanied by apoptosis induction of the MM cell lines after 7 days only, which, nevertheless, is in accordance with the literature [17].

In direct comparison to Dox, higher PIX concentrations were necessary to induce similar strong effects in myeloma cell lines as well as in primary patient cells. However, the improved toxicity profile of PIX [10, 11] argues for further studies with PIX.

We were able to demonstrate an impressive in vitro synergism between PIX and PAN with respect to their anti-proliferative features on myeloma cell lines. PAN is a potent, oral pan-deactylase inhibitor that increases acetylation of proteins involved in multiple oncogenic pathways [23]. It has been found to be synergistic with BTZ and Dex in preclinical studies of MM and has shown clinical activity in phase 1, 2, and 3 studies [24, 25]. The PAN-BTZ-Dex regimen demonstrated durable response in relapsed or refractory MM, including BTZ-refractory disease [26]. As PIX acts in a deferred way on mitosis [17] and PAN inhibits histone deacetylase [23], a cooperative biological mechanism centered around cell division-associated nuclear processes will be further investigated to increase our understanding of this striking phenomenon. The synergistic activity of PIX, PAN, and Dex might therefore be another treatment option, especially in very advanced MM (“penta-refractory”) patients.

Similar strong combinatorial activity was observed with Dox (in cell lines as well as in primary cells) and bendamustine. As at least bendamustine is a good therapeutic option in myeloma therapy—especially in renal failure—another interesting option to be investigated might thus be a bendamustine-PIX combination [27, 28].

In conclusion, PIX induced a strong and fast cytostatic effect in myeloma cell lines and induced apoptosis after a longer incubation period suggesting a putative role of PIX as part of possible combination therapies in relapsed/refractory MM and PCL patients in the framework of controlled clinical trials.

## Electronic supplementary material

Supplementary Figure 1 (DOCX 18 kb)
