# Supplementary material for: Pixantrone demonstrates significant in vitro activity against multiple myeloma and plasma cell leukemia
Source: Ann Hematol. 2019 Oct 18;98(11):2569–78. doi: 10.1007/s00277-019-03797-6 (PMC6848044; doi:10.1007/s00277-019-03797-6)
Supplement: Supplementary file 1 — (DOCX 18 kb) [file 277_2019_3797_MOESM1_ESM.docx]

**Supplemental Figure 1**

The effect of PIX on the proliferation of MM cell lines after a 72-hours incubation is shown. Mean proliferation + standard error of at least four experiments is depicted. Proliferation in the absence of PIX was set at 100%. Statistical significance was determined with the Wilcoxon test (*p<0.05 against the untreated control).
